# The NLRP6 protein is very faintly expressed in several normal and cancerous epithelial cells and may be confused with an unrelated protein

**DOI:** 10.1371/journal.pone.0279028

**Published:** 2023-01-20

**Authors:** Abdallah Mound, Gautier Goormachtigh, Fabrice Bray, Stéphanie Flament, Christian Rolando, Richard Ruez, Nathalie Martin, Amélie Decourcelle, Vanessa Dehennaut, Jean-Michel Saliou, Mathias Chamaillard, Corinne Abbadie

**Affiliations:** 1 Univ. Lille, CNRS, Inserm, CHU Lille, UMR9020-U1277—CANTHER—Cancer Heterogeneity Plasticity and Resistance to Therapies, Lille, France; 2 Univ. Lille, CNRS, USR 3290—MSAP—Miniaturization for Synthesis, Analysis & Proteomics, Lille, France; 3 Shrieking Sixties, Villeneuve-d’Ascq, France; 4 Univ. Lille, Inserm, U1003 –PhyCell–Laboratoire de Physiologie Cellulaire, Lille, France; 5 Univ. Lille, CNRS, Inserm, CHU Lille, Institut Pasteur de Lille, US 41 –UMS2014 –PLBS, Lille, France; University of the Pacific, UNITED STATES

## Abstract

Nod-Like Receptor Pyrin domain-containing protein 6 (NLRP6), a member of the Nucleotide-oligomerization domain-Like Receptor (NLR) family of proteins, assembles together with the ASC protein to form an inflammasome upon stimulation by bacterial lipoteichoic acid and double-stranded DNA. Besides its expression in myeloid cells, NLRP6 is also expressed in intestinal epithelial cells where it may contribute to the maintenance of gut homeostasis and negatively controls colorectal tumorigenesis. Here, we report that NLRP6 is very faintly expressed in several colon cancer cell lines, detected only in cytoplasmic small dots were it colocalizes with ASC. Consequently, it is very hardly detected by standard western-blotting techniques by several presently available commercial antibodies which, in contrast, highly cross-react with a protein of 90kDa that we demonstrate to be unrelated to NLRP6. We report here these results to caution the community not to confuse the 90kDa protein with the endogenous human NLRP6.

## Introduction

Nod-Like Receptor Pyrin domain-containing protein 6 (NLRP6), also called NALP6, NAVR, or PYPAF5, is a member of the Nucleotide-oligomerization domain-Like Receptor (NLR) family of proteins. NLRP6 is composed of an N-terminal Pyrin domain (PYD), a central Nucleotide-binding domain (NBD, also called the NACHT domain), and a C-terminal Leucine-Rich Region (LRR). NLRP6 is able to recruit the adaptor protein ASC (also called PYCARD) through PYD-PYD interactions, leading to large conformational changes and polymerization of ASC [1]. The recruitment of ASC via helical assembly solidifies NLRP6 condensates that become then able to recruit pro-caspase-1 and -11 via homotypic CARD-CARD interactions [2]. This results in the activation of the caspases and then in the proteolytic maturation and secretion of the pro-inflammatory IL-1β and IL-18 cytokines. All these homotypic interactions build what is called an inflammasome whose assembly is reinforced by further homotypic interactions between the NACHT and LRR domains of NLRP6 proteins. The NLRP6 inflammasome assembly is activated by various viral products or gram-positive and gram-negative bacteria, hence contributing to the innate immune response against microbial infections. Under steady-state conditions in the absence of ligand, it is though that NLRP6 inflammasome nucleation is prevented by the closed conformation of the LRR and NACHT domains [3, 4].

Besides its expression and function in monocytes [4, 5], NLRP6 was also shown to contribute to the maintenance of gut homeostasis and to proper wound healing of the colon, thereby acting as an epithelial-intrinsic tumor-suppressor gene [3, 6]. We and others previously reported that NLRP6 was primarily expressed in the intestine, mainly by colonic myofibroblasts and colonic epithelial cells [6]. There, it plays a role in controlling epithelial cell organization and proliferation after injury [6, 7]. In a model of colitis-associated colorectal cancer performed in *nlrp6*-/- mice, NLRP6 was shown to negatively controls colorectal tumorigenesis [6, 7]. Similarly, the over-expression of NLRP6 suppresses tumorigenicity in gastric cancer cells [8, 9]. However, it is not yet clear whether these homeostatic functions of NLRP6 in epithelial cells are dependent or not on the assembly of the NLRP6 inflammasome, with the downstream production of inflammatory cytokines.

Here, in order to further study the role of NLRP6 in human colon carcinogenesis, we analyzed its expression in a series of human colon carcinoma cell lines as well as in human primary cells. At some point during this study, we realized that the main protein detected in western-blotting experiments by several presently available commercial antibodies directed against human NLRP6 is not actually NLRP6. We show that the confusion is all the easier because NLRP6 is very faintly expressed in the investigated cells and therefore almost impossible to detect by standard western-blotting. We report here these results to caution the community presently working on NLRP6, and to incite the authors of previously published studies to re-examine their results.

## Materials and methods

### Cells

HCT116 (CCL-247), RKO, SW480 (CCL-228), HT29 (HTB-38) and HEK293T (CRL-3216) human cancer cell lines are from ATCC. The human normal fetal colonic cells CCD841 CoN (CRL-1790) are also from ATCC. OE19 (96071721) and A431 (85090402) cell lines are from ECACC. HPCECs are from Cell Biologics (H-6047; Lot#FF1415Y48FL/A). NHEKs K23FC and NHDFs F6MC1 are from PromoCell (Lot 401Z028.1and Lot 0080303.2 respectively).

### Cell culture

HCT116, RKO, SW480, HT29 and HEK293T cells were cultured in DNEM (High glucose, GlutaMax, Gibco, 6/065) supplemented with 10% fetal bovine serum. HPCECs were cultured in the Complete Epithelial Cell Medium from Cell Biologics (M6621) on dishes coated with a gelatin-based coating solution from Cell Biologics (6950). NHEKs K23FC were cultured in the KGM-Gold bulletkit medium (Clonetics) corresponding to a MCBD153 medium modified with 0.15mM calcium and supplemented with bovine pituitary extract, epidermal growth factor, insulin, hydrocortisone, transferrin, and epinephrine. NHDFs F6MC1 were cultured in the FGM-2 bulletkit medium (CC-3132, Lonza) consisting in Fibroblast Basal Medium (FBM, CC-3131 from Lonza) supplemented by 2% Fetal Bovine Serum (FBS), human Fibroblast Growth Factor (hFGF), Insulin at 5mg/mL, Gentamicin at 50μg/mL and Amphotericin at 50μg/mL. For HPCECs, NHEKs and NHDFs, the number of population doublings (PDs) was calculated at each passage by using the following equation: PD = log (number of collected cells/number of plated cells)/log2. All cell types were cultured in a water-saturated 5% CO_2_ atmosphere.

### Establishment of cells transiently overexpressing FLAG-NLRP6

The pcDNA3.1+/C-(K)-DYK vector plasmid expressing the full length NLRP6 protein (NM_138329.2) with a C-terminal FLAG epitope was constructed by GenScript (Clone ID: OHu21827D). The pcDNA3.1 empty plasmid was used as control. Cells were transiently transfected by a standard procedure using lipofectamine according to recommendations of the supplier (Lipofectamine® 2000 Reagent, Invitrogen). Proteins were extracted 48 or 72hrs post-transfection.

### Treatments

Cells were treated by ALLN (Calbiochem, 208719) at 25μM during 5hrs, Lactacystin (Sigma, L-6785) at 10μM during 5hrs, recombinant human IFN-β (Peprotech, 300-02BC) at 1ng/mL during 24hrs, recombinant human IFN-λ2 (Peprotech, 300-02K) at 2ng/mL during 24hrs, Poly(I:C) LMW (InvivoGen) at 10μg/mL during 24hrs, or with 20% of culture medium conditioned by NHDFs during the exponential growth phase or at the senescence plateau during 48hrs.

### Small interfering RNAs

siRNAs against NLRP6 (L-015334-01-0005, ON-TARGET plus Human NLRP6 (171389) siRNA-SMART pool, Dharmacon) or non-target siRNAs (D-001810-10-50, ON-TARGET plus non-targeting pool, Dharmacon) were transfected by a standard procedure using lipofectamine according to recommendations of the supplier (Lipofectamine® RNAiMAX Reagent, Invitrogen). Proteins were extracted 48hrs post-transfection. The NLRP6 target sequences were: GCGCCUACCGCUUCGUGAA, GGGCGCAGUUUGCCGAGAA, UCUCCGUGUCCGAGUACAA, GUGCAGACGGUCAGGGUAC. The non-target sequences were: UGGUUUACAUGUCGACUAA, UGGUUUACAUGUUGUGUGA, UGGUUUACAUGUUUUCUGA, UGGUUUACAUGUUUUCCUA.

### Total cell protein extracts

Cells were lysed in a 2X RIPA buffer containing 100mM Tris-HCl pH8, NaCl 300mM, EDTA 10mM, NP40 1%, plus protease inhibitors (Sigma S8830) added extemporaneously. The lysates were sonicated (22%, 2secs) and centrifugated at 14,000g for 15min at 4°C.

### Mouse colon protein extracts

C57BL/6 male mice purchased from Charles River Laboratories were maintained under a 12hrs light/dark cycle with a standard ad libidum diet. Twenty-six-week-old mice were sacrificed and colons were dissected, collected in FastPrep tubes (ref:6925100, MP Biomedicals) and immediately frozen at -80°C. Proteins were further extracted in RIPA buffer as done for in vitro cultured cells.

### Western-blotting

Proteins were separated by Sodium Dodecyl Sulfate-polyacrylamide gel electrophoresis (SDS-PAGE) and transferred on nitrocellulose membranes (88018, Thermo Fischer Scientific). Membranes were blocked with 5% non-fat dried milk or 5% BSA in PBS. Then, membranes were incubated overnight with the primary antibody at the dilution recommended by the manufacturer: anti-NLRP6 (AP13529a, ABGENT), anti-NLRP6 (N) (R3097-1, Abiocode), anti-NLRP6 (C2) (R3097-3, Abiocode), anti-NLRP6 (NALP6) (ABF29, Millipore), anti-NLRP6 (A15628, ABclonal), anti-FLAG (M2) (Sigma, F1804), anti-tubulin-α (Sant Cruz Biotechnology), and anti-GAPDH (sc-32233, Santa Cruz Biotechnology). Secondary antibodies used were: anti-mouse and anti-rabbit peroxidase conjugated (respectively 715-035-151, 711-035-152, Jackson-immuno research Laboratories). The peroxidase activity was revealed using an ECL kit (RPN2106, Amersham Biosciences) or SuperSignal West Dura Extended Duration Substrate (34076, Thermo Fischer Scientific).

### Immunoprecipitation

Cells were rinsed in cold PBS and lysed in cold IPH buffer (50mM Tris [pH 8], 150mM NaCl, 5mM EDTA, 0.5% NP40, and a protease inhibitor cocktail [Sigmafast]). Cell lysates were cleared by centrifugation (14,000g, 4°C, 15 min). The supernatants were pre-cleared by incubation with proteinA/G sepharose beads (Amersham Biosciences) 1hr at 4°C and the supernatants were collected after a centrifugation at 8,000g for 1min. The supernatants were then incubated overnight at 4°C with the anti-NLRP6 antibody and then the A/G beads were added for an additional 1hr. The beads were washed four times with IPH buffer. Bound proteins were eluted by boiling 5min in Laemmli sample buffer and analyzed by SDS-PAGE as above.

### Immunofluorescence

Cells were fixed on ice with cold acetone/methanol (v/v) for 10min and washed with PBS. Unspecific sites were blocked by incubation at room temperature with 1% BSA in PBS. Then, epitopes were detected by co-incubation overnight at 4°C with antibodies against NLRP6 (ABGENT AP13529a) and ASC (B-3) (Santa Cruz Biotechnology, sc-514414). After washing with PBS, cells were co-incubated with the secondary antibodies (Life Technologies, A21206 and A-21202) followed by nuclear staining with 300nM DAPI (Life technologies, D1306). The results were analyzed on an AxioImager Z1-Apotome from Zeiss.

### Mass spectrometry analysis

Six bands at the positions corresponding to positivity in western-blot analysis using the anti-NLRP6 antibody from ABGENT were excised from gel, cut in cubes of approximately 1mm edge length and deposited into Eppendorf® tubes. Proteins in the gel bands were digested according to the protocol previously described elsewhere [10, 11]. Briefly, the cubes were washed, reduced with DTT, alkylated with IAA and digested with trypsin solution (Promega V511A). Peptides were extracted and dried in a vacuum centrifuge SpeedVac™ Concentrator (Eppendorf^TM^ Concentrator Plus, Eppendorf). Prior to LC-MS/MS analysis, peptides were dissolved in 10μL of 0.1% (v/v) formic acid. LC-MS/MS analyzes were performed on an Orbitrap Q Exactive plus Mass Spectrometer hyphenated to a U3000 RSLC Microfluidic HPLC System (ThermoFisher Scientific). One μl of peptide mixture diluted in solution A (5% v/v acetonitrile and 0.1% formic acid) was injected. Peptides were concentrated on Acclaim PepMap100 C18 pre-column (5μm, 300μm i.d. × 5mm) (ThermoFisher Scientific), then were separated on a C18 Acclaim PepMap100 C18 reversed phase column (3μm, 75mm ID × 500mm) (ThermoFisher Scientific), using a linear gradient (5–40%) of solution B (75% ACN and 0.1% formic acid) using a flow-rate of 250nL min-1. LC runs were acquired in positive ion mode with MS scans from *m/z* 350 to 1,500 in the Orbitrap mass analyzer with a 70,000 resolution for MS and 35,000 resolution for MS/MS.

Raw files from in-gel digestion were analyzed using Proteome Discoverer™ (Thermo Scientific, version 2.2). SEQUEST algorithm was used for database searches with the UniProtKB/Swiss-Prot reviewed *Homo sapiens* database (*Homo sapiens*, 2021_03, Sequences: 20,386) and NLRP6 sequence NM_138329.2 using the following search parameters. Trypsin was selected as the digestion enzyme with 3 allowed missed cleavages. The error on MS precursor was set to 10ppm (parts per million) and that on MS/MS fragment was set to 0.02Da. Methionine and proline oxidation, lysine acetylation, deamidation of asparagine or glutamine (NQ) and serine/threonine phosphorylation were set as dynamic modifications and carbamidomethyl cysteine was set as fixed modification. Proteins were identified with a minimum of 2 matching peptides including 1 unique peptide, and a SEQUEST score higher than 20.

### Ethics statement

The part of this study involving mice has received a favorable statement from our ethic committee (CEEA 75. Nord Pas-de-Calais, France) and was authorized by the Ministère de l’éducation nationale, de l’enseignement supérieur et de la recherche under the number APAFIS#1879–2018121918307521.

## Results

### The NLRP6 protein is faintly expressed in several human and mouse cell types

**Fig 1:**
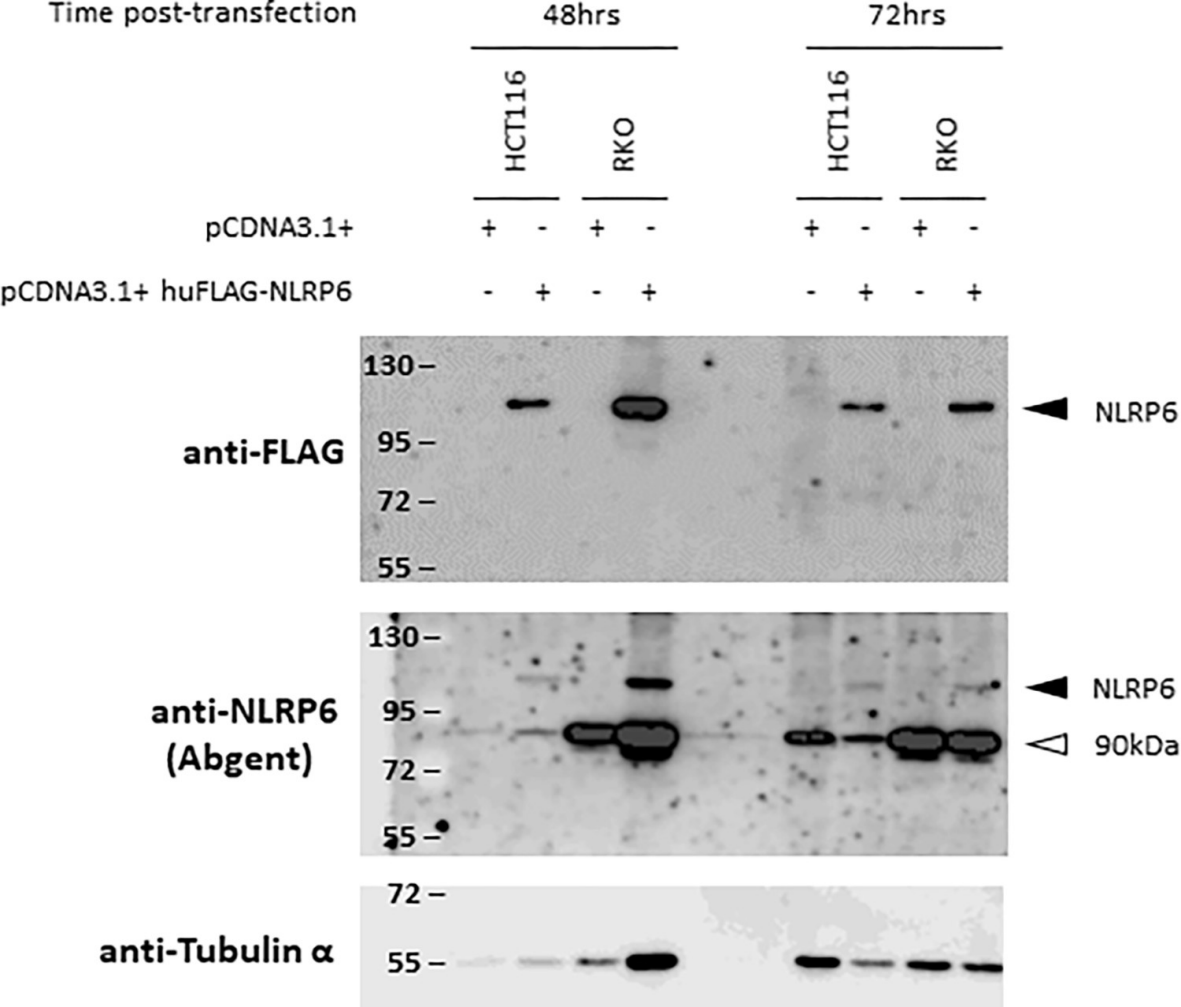
The endogenous NLRP6 expression is not detected in HCT116 and RKO cancer cell lines by western-blotting using an antibody from ABGENT. Western-blot analysis of extracts of HCT116 and RKO cells made 48 or 72hrs after transfection with a pCDNA3.1+ vector either empty or encoding the human full-length FLAG-NLRP6 protein. The migration position of NLRP6 is indicated with a dark arrowhead. The white arrowhead points a non-specific band at 90kDa.

In order to further study the role of NLRP6 in colorectal carcinogenesis, we transiently overexpressed the full length NLRP6 protein (NM_138329.2) with a C-terminal FLAG epitope in the human colon cancer cell lines HCT116 and RKO. We performed a western-blot analysis 48 and 72hrs post-transfection using both anti-FLAG and anti-NLRP6 antibodies. The results with the anti-FLAG antibody indicate that FLAG-NLRP6 was indeed expressed in both cell lines, at the apparent MW of 110kDa. The same band was also detected by the anti-NLRP6 antibody. However, we were unable to detect any endogenous NLRP6 expression with the anti-NLRP6 antibody (Fig 1).

**Fig 2:**
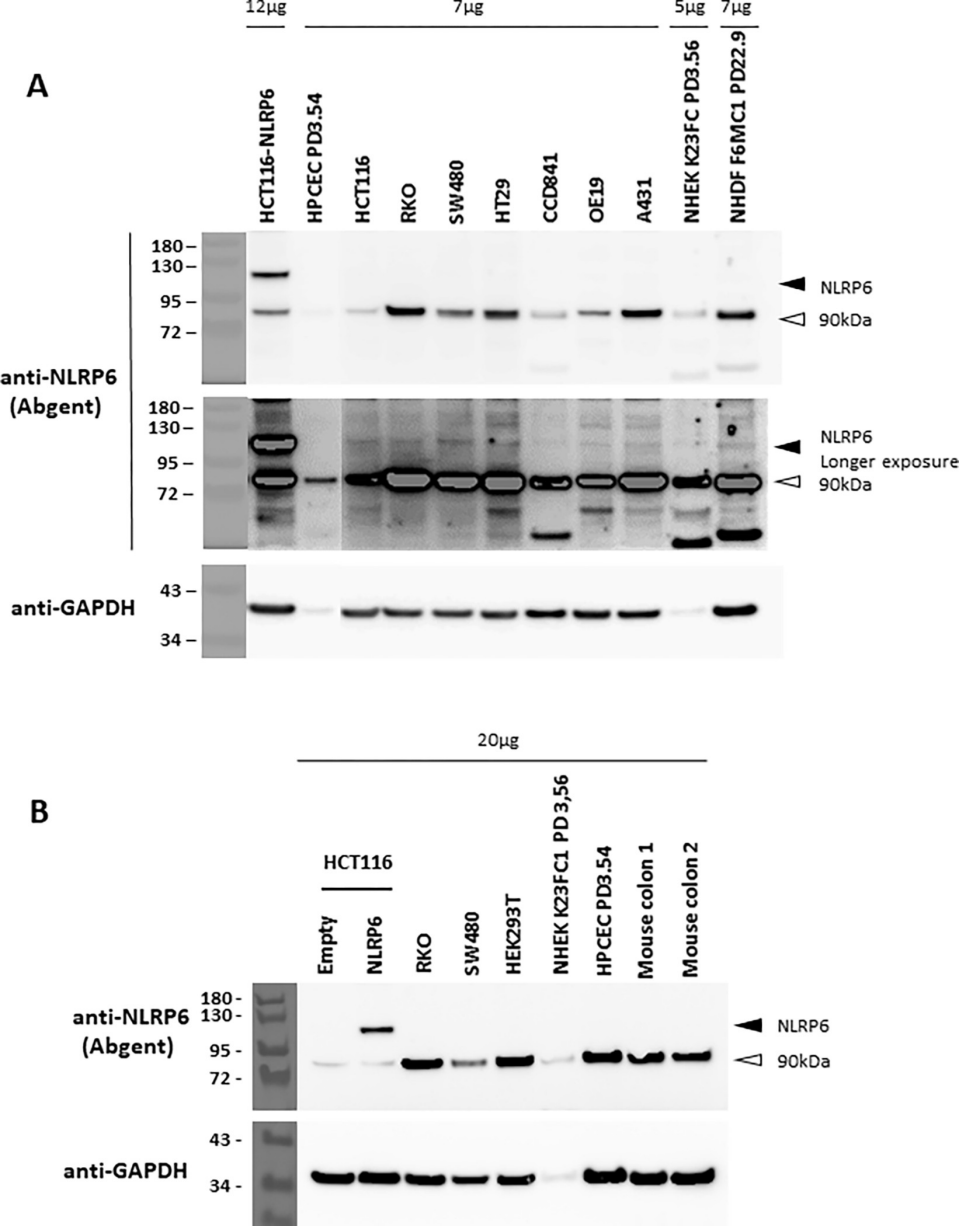
The endogenous NLRP6 expression is barely detected in several human and mouse cell types by western-blotting using an antibody from ABGENT. (A) Western-blot analysis of extracts of HCT116 cells transiently transfected by the FLAG-NLRP6-expressing pCDNA3.1+ vector, and of a series of various non-transfected cell lines. The upper image corresponds to a short exposure and the lower image to a longer exposure. (B) Western-blot analysis of the same extracts as in A and total mouse colon extracts. The migration position of NLRP6 is indicated with a dark arrowhead. The white arrowhead points a non-specific band et 9OkDa. The amount of loaded total proteins is indicated for each lane.

In order to establish whether this poor endogenous NLRP6 expression was also true for other cell types, we performed western-blot analyses on a variety of cell types: two other human colon cancer cell lines (SW480 and HT29), the human embryonic kidney cell line HEK293T, human normal fetal colonic cells (CCD841), human normal adult colonic epithelial cells (HPCECs), the human adenocarcinoma of esophageal gastric junction cell line OE19, the human epidermal carcinoma cell line A431, human primary epidermal keratinocytes (NHEKs K23FC), and human primary dermal fibroblasts (NHDFs F6MC1). We were unable to detect the endogenous NLRP6 protein in any of these normal or cancerous cell types by this standard western-blotting technique, the specific band being only detected after long exposures, but not above the general background (Fig 2A and 2B). Since NLRP6 was often studied in mouse models, we also tried to detect it in mouse colonic extracts. Again, we were unable to detect any endogenous NLRP6 proteins in these extracts by standard western-blot (Fig 2B).

In order to favor the detection of endogenous NLRP6, we attempted to inhibit its possible degradation. For that, HCT116 and SW480 cells were treated with the cysteine protease inhibitor *N-*acetyl-I-leucyl-I-leucyl-I-norleucinal (ALLN) or with the proteasome inhibitor Lactacystin. However, NLRP6 was still undetectable by western-blotting after these treatments (S1A Fig). Conversely, we tried to increase its expression by several known inducers [3]. HCT116, RKO and SW480 cells were treated with Interferon (IFN)-λ2, IFN-β, Poly(I:C), or by a culture medium conditioned by NHDFs in replicative senescence which contains high levels of various inflammatory cytokines. However, the expression of the endogenous NLRP6 was still undetected by western-blotting (S1B Fig).

**Fig 3:**
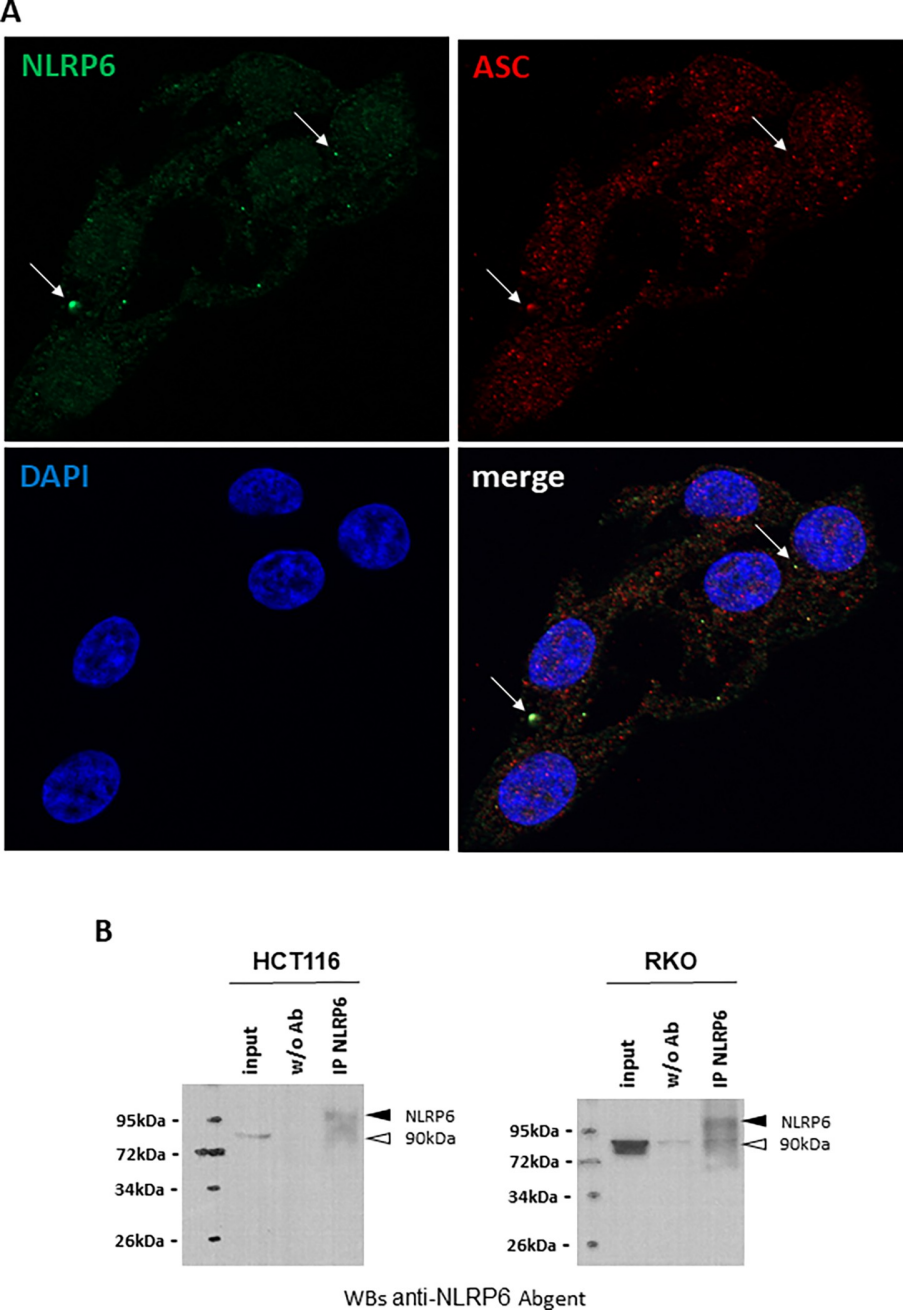
The endogenous NLRP6 protein is present only in cytoplasmic dots also containing the ASC protein. (A) Immunofluorescence microscopy images of HCT116 cells showing the colocalization of NLRP6 and ASC in small cytoplasmic dots (white arrows). (B) Immunoprecipitation of NLRP6 in HCT116 and RKO cell extracts followed by an analysis with anti-NLRP6 antibody from ABGENT. NLRP6 is indicated with a dark arrowhead. The white arrowhead points a non-specific doublet at 90kDa.

Finally, we performed a detection of NLRP6 by immunofluorescence on HCT116 cells. To ensure the specificity of the staining, we co-detected ASC. The results show that NLRP6 is present at maximum in one small cytosolic dot per cell, where it colocalizes with ASC (Fig 3A), indicating that these dots are probably inflammasomes, and explaining why the endogenous NLRP6 is hardly detected in western-blots of total cellular extracts. We then performed an immunoprecipitation with an anti-NLRP6 antibody, followed by a western-blot detection with the same antibody, in HCT116 and RKO cells. This technique allowed us to detect the protein, although hardly, confirming its poor expression in these cells (Fig 3B).

### Several anti-NLRP6 commercial antibodies strongly cross-react with an unrelated protein of 90kDa

In all our western-blots, the anti-NLRP6 antibody from ABGENT that we used strongly detected a band at about 90kDa. We first thought this band was NLRP6 for several reasons: (i) because it was designed as NLRP6 in the data sheet given by ABGENT, despite its wrong molecular weight, (ii) because the quantity of this protein of 90kDa was differential in different cell types (S2A Fig), (iii) because its expression was differentially induced at senescence in different cell types (S2A Fig), and (iv) because its expression was induced by IFNγ (S2B Fig).

In some of our western-blots, according to the percentage of acrylamide and/or to the duration of the migration, we could see that the ABGENT antibody actually recognized a doublet of bands of close molecular weight around 90kDa. The two bands of the doublet were differentially expressed in different cell types, with the upper band most often more expressed than the lower one (S1A and S2A Figs).

Because of the cleanliness of the signal in the western-blots, we made the hypothesis that the 90kDa protein(s) could correspond to a truncated form of NLRP6. A search in the gene data base (https://www.ncbi.nlm.nih.gov/gene) revealed the existence of a predicted NLRP6 isoform X1 (XP_016872742.1) of 730 amino acids, devoid of PYD, which could match in term of molecular weight (S3 Fig). This truncated form could theoretically be recognized by the ABGENT polyclonal antibody we used (ABGENT AP13529a) since the immunogen is an epitope localized just upstream of the NACHT domain, still present in the predicted truncated form of NLRP6 (S3A and S4A Figs).

To specifically test this hypothesis, we first performed western-blotting experiments with other commercial antibodies directed either against an epitope localized in the PYD (Abiocode R3097-1) which should not react with the 90kDa doublet, or in the LRR domain (Abiocode R3097-3), which should (S4 Fig). The Abiocode antibody directed against the C term domain did detect FLAG-NLRP6 in HCT116 cells and the endogenous protein in HCT116 and RKO cells, as well as the 90kDa protein. However, and unfortunately, the Abiocode antibody directed against the PYD did not recognize either the endogenous or FLAG-NLRP6 or the 90kDa doublet, ruling out any conclusion (S4B Fig). Notice that although the Abiocode antibody directed against the C-term domain detects the endogenous NLRP6, it cannot be used easily to study NLRP6 because it cross-reacts with another band of very close molecular weight which is also recognized by the Abiocode antibody directed against the PYD (S4B Fig). We also tried a third antibody from Millipore (ABF29, lot2775315), directed against the NACHT domain. This antibody also faintly recognized the 90kDa doublet and other aspecific bands but not a band at the right molecular weight (S4B Fig). And finally, we tested an antibody from ABclonal (A15628) directed against a peptide localized in between the PYD and the NACHT domains. This antibody was able to recognize the overexpressed FLAG-NLRP6, but did not detect the endogenous NLRP6; it also cross-reacted with the 90kDa doublet (S4B Fig).

To further determine if the 90kDa protein could correspond to a truncated form of NLRP6, we performed siRNA experiments. We used a siRNAs Smartpool from Dharmacon, made of 4 siRNAs spanning the NLRP6 sequence. These siRNAs did decrease the expression of FLAG-NLRP6 but did not affect the 90kDa band, indicating that this protein does not share sequences with NLRP6 (S5 Fig).

We finally wanted to determine whether the 90kDa doublet protein could correspond to another protein of the NLRP family or to another protein presenting a PYD and/or a NACHT domain. To investigate this point, we performed a mass spectrometry analysis of HCT116 cells overexpressing FLAG-NLRP6 versus HCT116 control cells, as well as non-transfected RKO cells which highly expressed the 90kDa doublet. We collected gel fragments in each line of the SDS-PAGE gel both at the migration position of the FLAG-NLRP6 and at that of the 90kDa doublet (S6 Fig). The mass spectrometry analysis of the gel fragments confirmed the presence of NLRP6 in the expected gel fragment (fragment 4) (S4 Table) but not in the others (S1–S3, S5 and S6 Tables). The most abundant proteins in the gel fragments at the 90kDa level were HSP90 alpha and HSP90 alpha isoform 2 (respectively coded by HSP90AB1 and HSP90AA1). None of the other identified proteins at this position were related to NLRP6 (S7 Table). In support to the notion that the anti-NLRP6 could cross-react with a HSP90 protein, a sequence alignment using the LALIGN software (https://www.ebi.ac.uk/Tools/psa/lalign/) revealed that the first 10 amino acids of the synthetic peptide used as immunogen by ABGENT share 50% identity (90% similarity) with HSP90 alpha, and the last 20 amino acids 25% identity (83.3% similarity) (S1 File).

## Discussion

The expression of NLRP6 was initially described to be restricted to granulocytes and T-cells [12], but it was further shown to be expressed in monocytes, dendritic cells, and macrophages, as well as in epithelial cells and myofibroblasts of the intestine [3]. Here we show that in various human normal and cancerous cell types the expression of NLRP6 is very low, almost undetectable by standard western-blotting experiments. The endogenous NLRP6 was also barely detected in colonic extracts of WT mice using the ABGENT antibody. Immunofluorescent experiments on HCT116 cells revealed that the protein is present only in small dots, at most one per cell, co-localizing there with ASC, suggesting these dots are inflammasomes. The interaction of NLRP6 with ASC was already demonstrated by others, using a model of overexpression of FLAG-NLRP6, MYC-Caspase 1 and HA-ASC in HEK293-T cells [13]. Here we confirmed this interaction by deconvoluted microscopy and showed it for the first time between the endogenous NLRP6 and the endogenous ASC proteins in intestinal epithelial cancer cells.

Most of the commercial antibodies against NLRP6 we used did recognize FLAG-NLRP6, as well as the endogenous NLRP6 after immunoprecipitation or by immunofluorescence. Therefore, their quality is not to blame. However, and unfortunately, they highly cross-react with a doublet band migrating in SDS-PAGE at 90kDa that can be confused with NLRP6, a confusion that was done by the antibody supplier ABGENT itself in its datasheet. We are concerned that the mistake may have been made in several published studies, and we recommend to be cautious with conclusions drawn from western-blotting results where the position of the molecular weight markers is not given.

We demonstrate in this study that the doublet protein cross-reacting with the anti-NLRP6 antibody is neither a truncated form of NLRP6 nor a related protein possessing a PYD or a NACHT domain. Mass spectrometry analysis and sequence alignments suggest that the doublet could be the HSP90 alpha and HSP90 alpha isoform 2 proteins.

## Supporting information

S1 Fig The endogenous NLRP6 expression is not detected in human colon cancer cell lines after several treatments by western-blotting using an antibody from ABGENT. (A) Western-blot analysis of extracts of HCT116 or SW480 cells treated during 5hrs with 25μM ALLN or 10μM Lactacystin. (B) Western-blot analysis of extracts of HCT116, RKO or SW480 treated by recombinant human IFN-β at 1ng/mL during 24hrs, recombinant human IFN-λ2 at 2ng/mL during 24hrs, Poly(I:C) LMW at 10μg/mL during 24hrs, or with 20% of culture medium conditioned by NHDFs during the exponential growth phase (CM ExpG NHDFs) or at the senescence plateau (CM Sen NHDFs) during 48hrs. The migration position of NLRP6 is indicated with a dark arrowhead. The white arrowhead points a non-specific doublet at 90kDa.
(TIF)

S2 Fig The expression of the 90kDa doublet protein is inducible. (A) Western-blot analysis of extracts of NHEKs and NHDFs during the exponential growth phase (ExpG) or at the senescence plateau (Sen), and of HCT116, RKO and SW480 cells. (B) Western-blot analysis of extracts of HCT116 and RKO cells treated with recombinant human IFN-γ at 1ng/mL during 24hrs or Poly(I:C). The expected position of NLRP6 is indicated with a dark arrowhead. The white arrowhead points a non-specific doublet at 90kDa whose expression varies in these different conditions.
(TIF)

S3 Fig Predicted NLRP6 short isoform. (A) Schematic representation of the structure of the NLRP6 gene and of the encoded protein. (B) Representation of the different transcripts. The predicted transcript devoid of the PYD is highlighted with a red arrow.
(TIF)

S4 Fig Specificity of a few anti-NLRP6 commercial antibodies. (A) Schematic representation of the structure of the NLRP6 protein with the position of the epitopes against which the antibodies used in B are directed. (B) Western-blot analysis of extracts of HCT116 cells transiently transfected by the empty or FLAG-NLRP6-expressing pCDNA3.1+ vectors and of non-transfected RKO cells with antibodies from Abiocode and ABGENT. NLRP6 is indicated with a dark arrowhead. The white arrowhead points the non-specific doublet at 90kDa. The red star underlines another non-specific band migrating very close to NLRP6. (C) Western-blot analysis with antibodies from Millipore and ABclonal of extracts of non-transfected HPCEC cells, non-transfected RKO cells and HCT116 cells transiently transfected by the FLAG-NLRP6-expressing pCDNA3.1+ vectors and then transfected with siRNAs against NLRP6 or control siRNAs of non-transfected RKO cells. NLRP6 is indicated with a dark arrowhead. The white arrowhead points the non-specific doublet at 90kDa.
(TIF)

S5 Fig The 90kDa protein is unsensitive to siRNAs directed against NLRP6. Western-blot analysis with the anti-NLRP6 antibody from ABGENT of extracts of HCT116 cells transiently transfected with the empty or the FLAG-NLRP6-expressing pCDNA3.1+ vector and further transfected with siRNAs against NLRP6. NLRP6 is indicated with a dark arrowhead. The white arrowhead points the non-specific doublet at 90kDa.
(TIF)

S6 Fig Preparation of samples for mass spectrometry analysis. Right: photograph of the SDS-PAGE gel in which gel fragments were collected and numbered for further Mass spectrometry analysis. Left: image of a representative western-blot analysis using the anti-NLRP6 from ABGENT of the expression of NRLP6 and of the 90kDa protein in the extracts used for the Mass Spectrometry analysis. NLRP6 is indicated with a dark arrowhead. The white arrowhead points the non-specific doublet at 90kDa.
(TIF)

S1 Raw images (TIF)

S2 Raw images (TIF)

S3 Raw images (TIF)

S4 Raw images (TIF)

S5 Raw images (TIF)

S6 Raw images (TIF)

S7 Raw images (TIF)

S8 Raw images (TIF)

S9 Raw images (TIF)

S10 Raw images (TIF)

S11 Raw images (TIF)

S12 Raw images (TIF)

S13 Raw images (TIF)

S14 Raw images (TIF)

S1 Table Content of gel fragment 1 in NLRP6 peptides. (XLSX)

S2 Table Content of gel fragment 2 in NLRP6 peptides. (XLSX)

S3 Table Content of gel fragment 3 in NLRP6 peptides. (XLSX)

S4 Table Content of gel fragment 4 in NLRP6 peptides. (XLSX)

S5 Table Content of gel fragment 5 in NLRP6 peptides. (XLSX)

S6 Table Content of gel fragment 6 in NLRP6 peptides. (XLSX)

S7 Table The most abundant proteins in gel fragment 1 and 5. (XLSX)

S1 File Percentage of identity and similarity between the sequence of the NLRP6 peptide used as immunogen by ABGENT. The sequence of the synthetic peptide corresponding to amino acids 168 to 197 of the human NLRP6 used as immunogen was aligned against the sequence of human HSP90 alpha.
(TXT)
