# Supplementary figures and images for: The NLRP6 protein is very faintly expressed in several normal and cancerous epithelial cells and may be confused with an unrelated protein
Source: PLoS One. 2023 Jan 20;18(1):e0279028. doi: 10.1371/journal.pone.0279028 (PMC9858803; doi:10.1371/journal.pone.0279028)

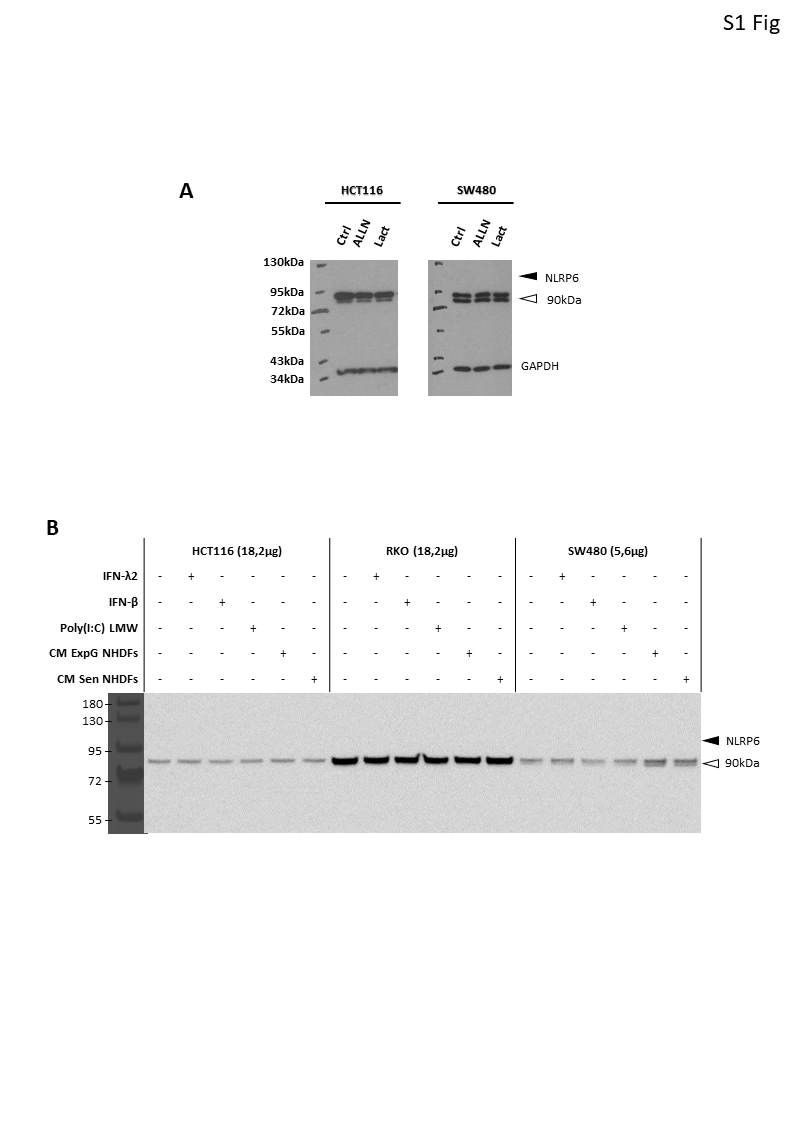

Supplement: S1 Fig — (A) Western-blot analysis of extracts of HCT116 or SW480 cells treated during 5hrs with 25μM ALLN or 10μM Lactacystin. (B) Western-blot analysis of extracts of HCT116, RKO or SW480 treated by recombinant human IFN-β at 1ng/mL during 24hrs, recombinant human IFN-λ2 at 2ng/mL during 24hrs, Poly(I:C) LMW at 10μg/mL during 24hrs, or with 20% of culture medium conditioned by NHDFs during the exponential growth phase (CM ExpG NHDFs) or at the senescence plateau (CM Sen NHDFs) during 48hrs. The migration position of NLRP6 is indicated with a dark arrowhead. The white arrowhead points a non-specific doublet at 90kDa. (TIF) [file pone.0279028.s001.TIF]

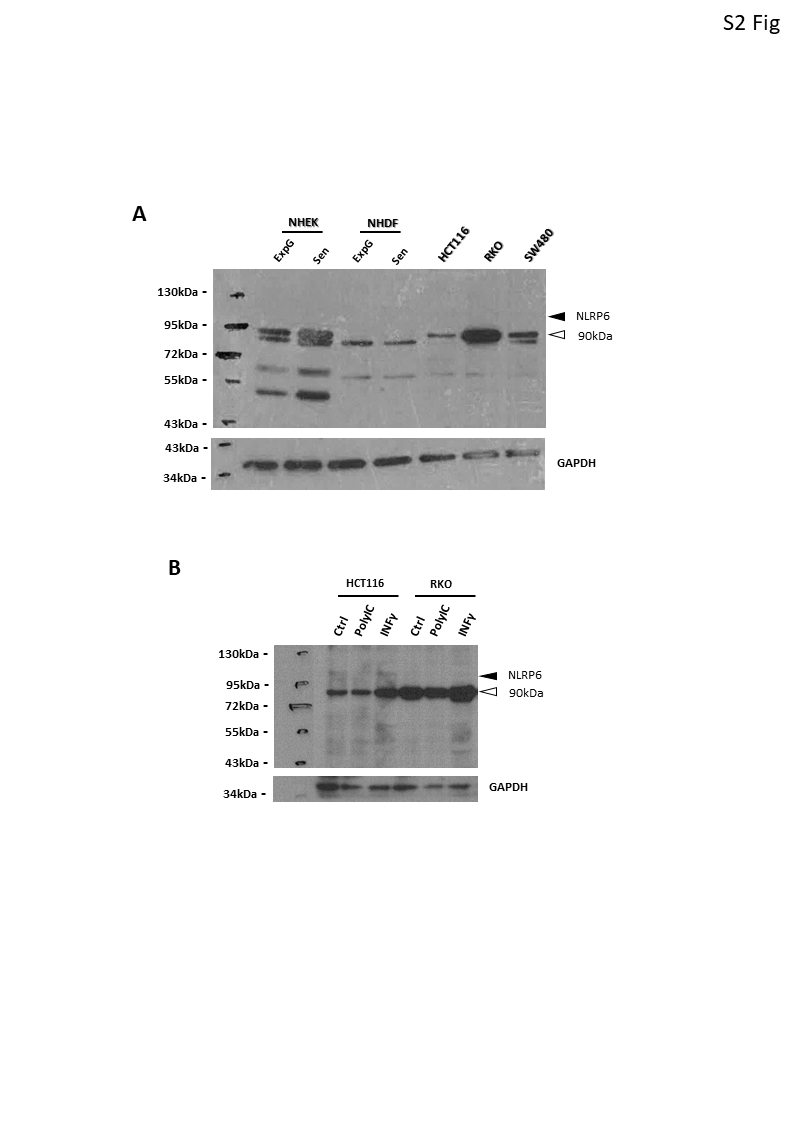

Supplement: S2 Fig — (A) Western-blot analysis of extracts of NHEKs and NHDFs during the exponential growth phase (ExpG) or at the senescence plateau (Sen), and of HCT116, RKO and SW480 cells. (B) Western-blot analysis of extracts of HCT116 and RKO cells treated with recombinant human IFN-γ at 1ng/mL during 24hrs or Poly(I:C). The expected position of NLRP6 is indicated with a dark arrowhead. The white arrowhead points a non-specific doublet at 90kDa whose expression varies in these different conditions. (TIF) [file pone.0279028.s002.TIF]

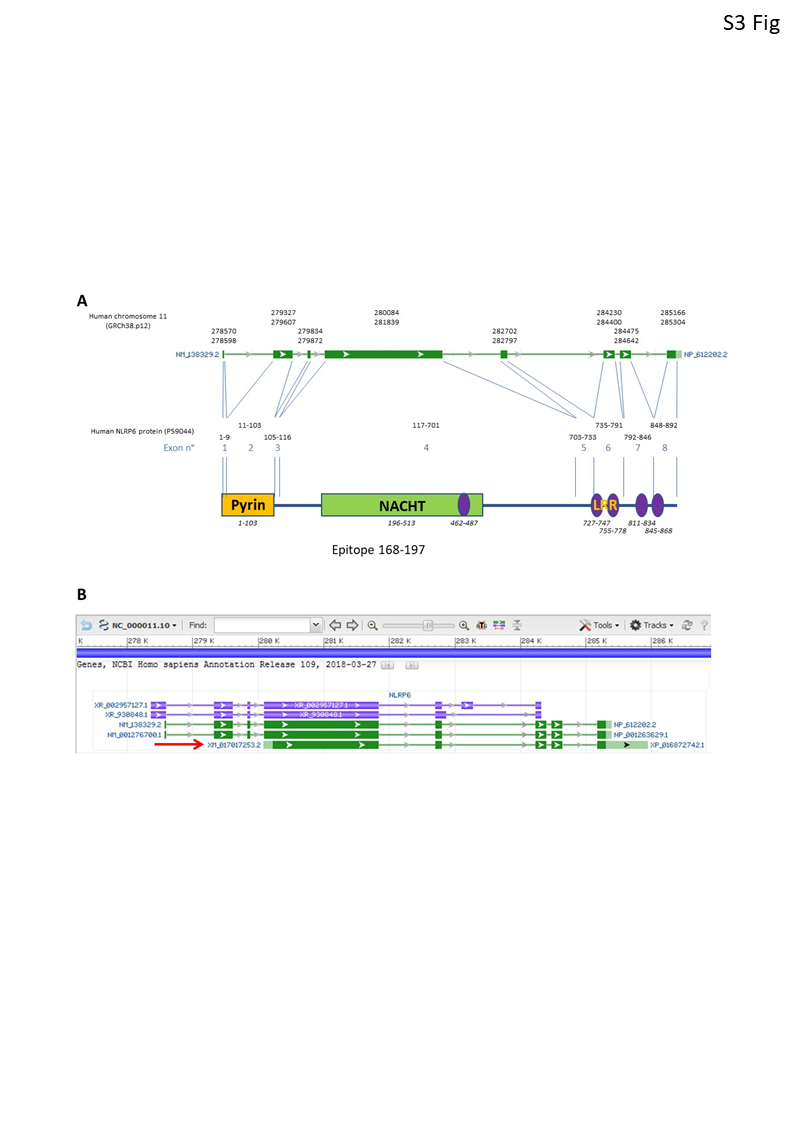

Supplement: S3 Fig — (A) Schematic representation of the structure of the NLRP6 gene and of the encoded protein. (B) Representation of the different transcripts. The predicted transcript devoid of the PYD is highlighted with a red arrow. (TIF) [file pone.0279028.s003.TIF]

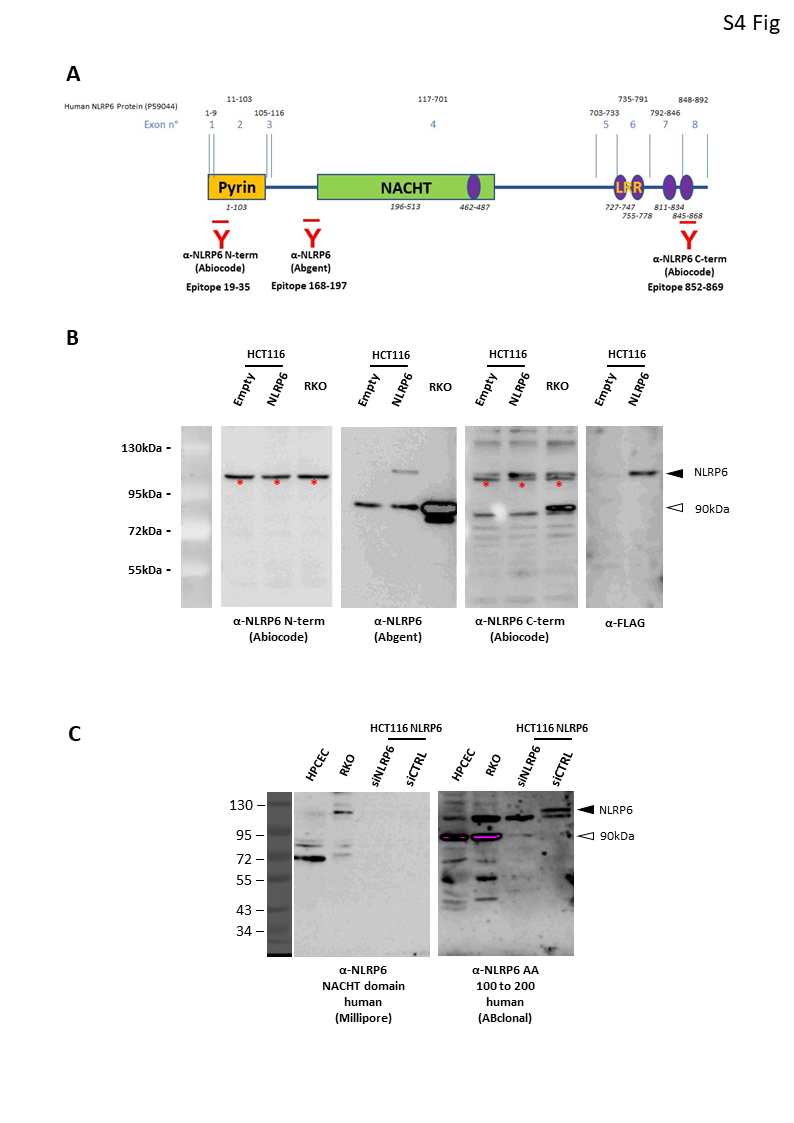

Supplement: S4 Fig — (A) Schematic representation of the structure of the NLRP6 protein with the position of the epitopes against which the antibodies used in B are directed. (B) Western-blot analysis of extracts of HCT116 cells transiently transfected by the empty or FLAG-NLRP6-expressing pCDNA3.1+ vectors and of non-transfected RKO cells with antibodies from Abiocode and ABGENT. NLRP6 is indicated with a dark arrowhead. The white arrowhead points the non-specific doublet at 90kDa. The red star underlines another non-specific band migrating very close to NLRP6. (C) Western-blot analysis with antibodies from Millipore and ABclonal of extracts of non-transfected HPCEC cells, non-transfected RKO cells and HCT116 cells transiently transfected by the FLAG-NLRP6-expressing pCDNA3.1+ vectors and then transfected with siRNAs against NLRP6 or control siRNAs of non-transfected RKO cells. NLRP6 is indicated with a dark arrowhead. The white arrowhead points the non-specific doublet at 90kDa. (TIF) [file pone.0279028.s004.TIF]

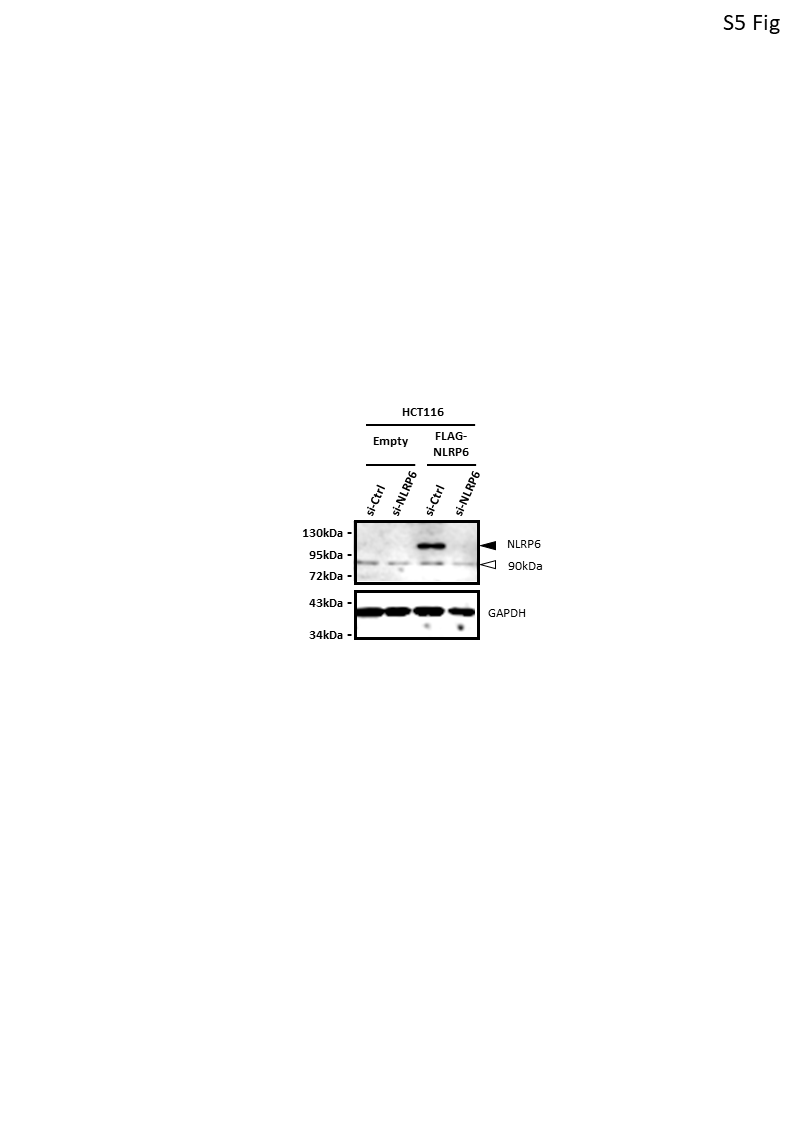

Supplement: S5 Fig — Western-blot analysis with the anti-NLRP6 antibody from ABGENT of extracts of HCT116 cells transiently transfected with the empty or the FLAG-NLRP6-expressing pCDNA3.1+ vector and further transfected with siRNAs against NLRP6. NLRP6 is indicated with a dark arrowhead. The white arrowhead points the non-specific doublet at 90kDa. (TIF) [file pone.0279028.s005.TIF]

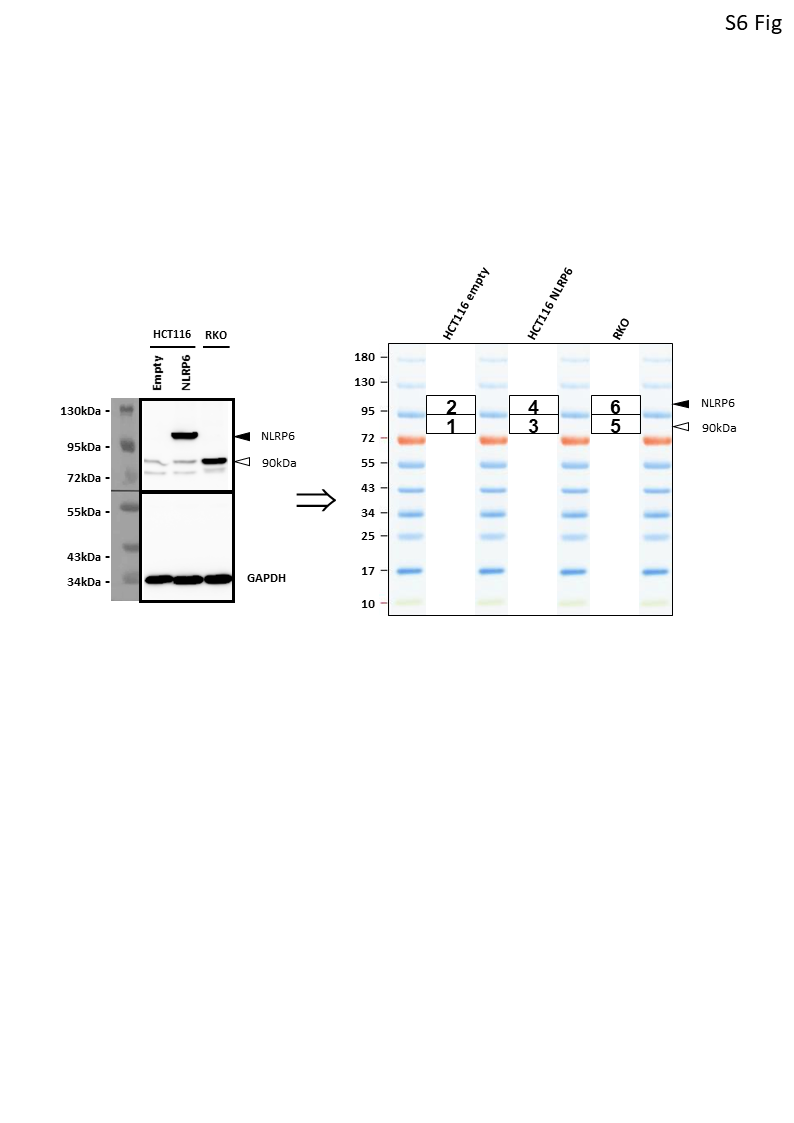

Supplement: S6 Fig — Right: photograph of the SDS-PAGE gel in which gel fragments were collected and numbered for further Mass spectrometry analysis. Left: image of a representative western-blot analysis using the anti-NLRP6 from ABGENT of the expression of NRLP6 and of the 90kDa protein in the extracts used for the Mass Spectrometry analysis. NLRP6 is indicated with a dark arrowhead. The white arrowhead points the non-specific doublet at 90kDa. (TIF) [file pone.0279028.s006.TIF]

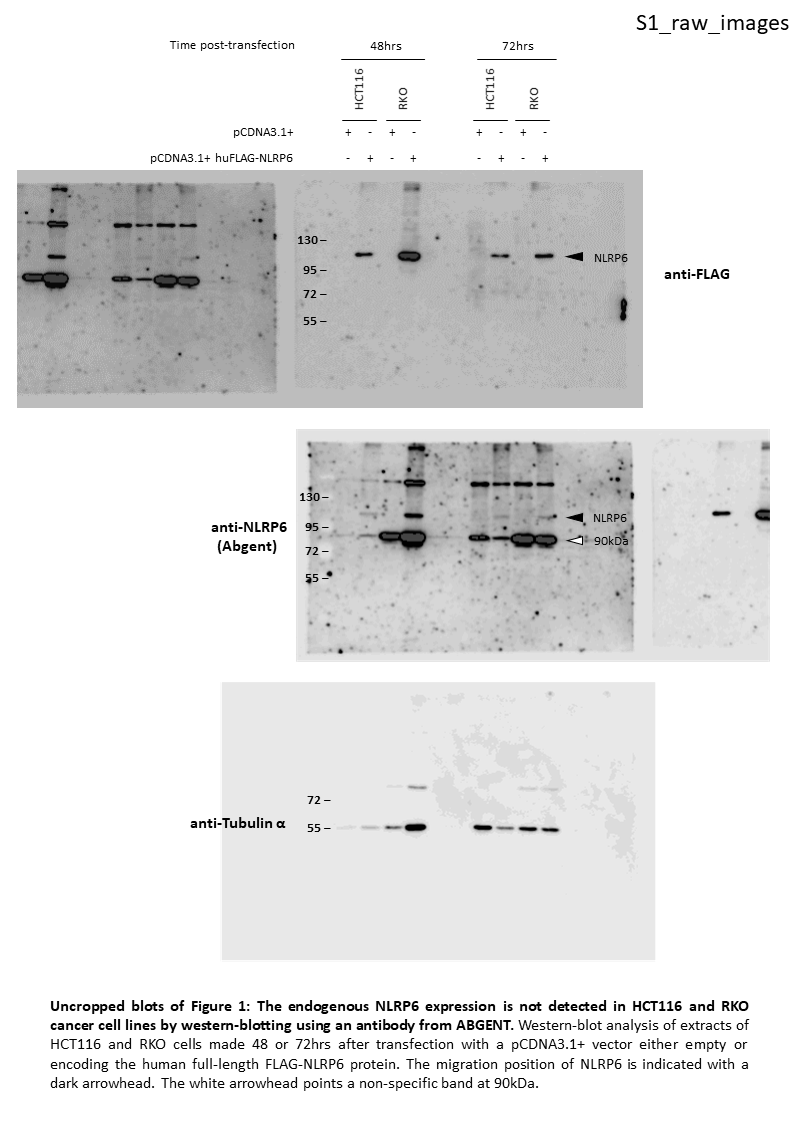

Supplement: S1 Raw images — (TIF) [file pone.0279028.s007.TIF]

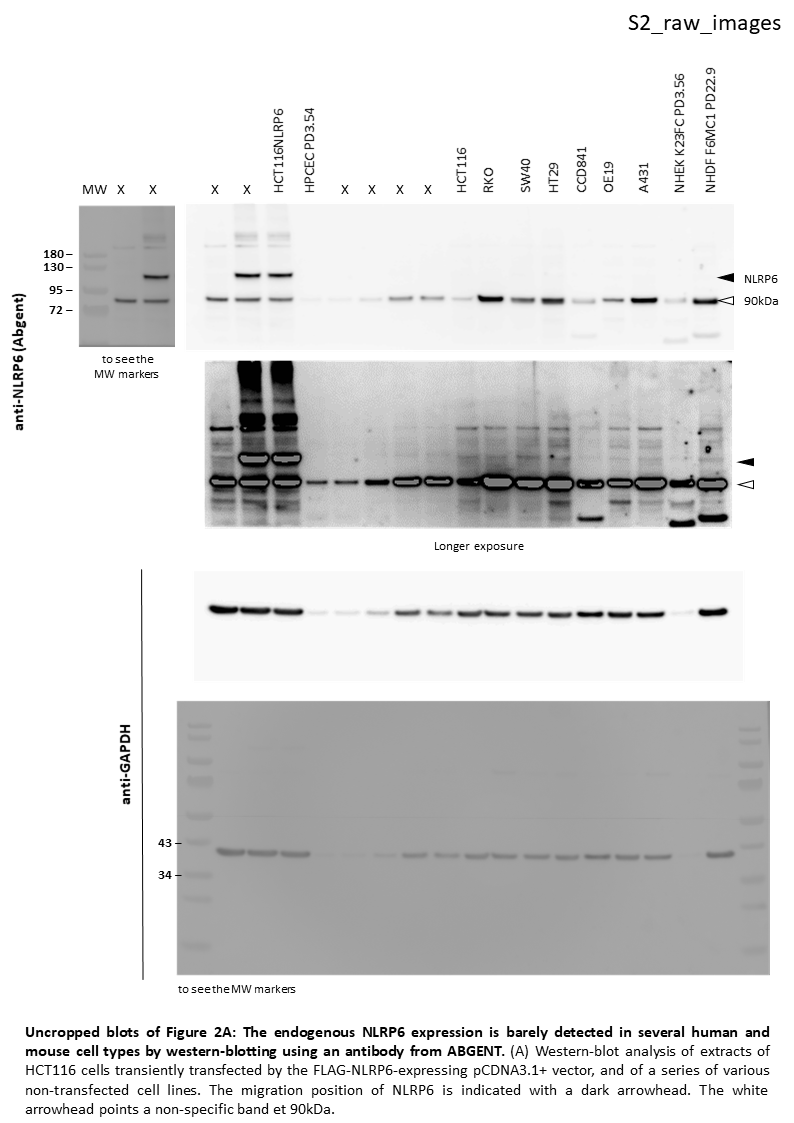

Supplement: S2 Raw images — (TIF) [file pone.0279028.s008.TIF]

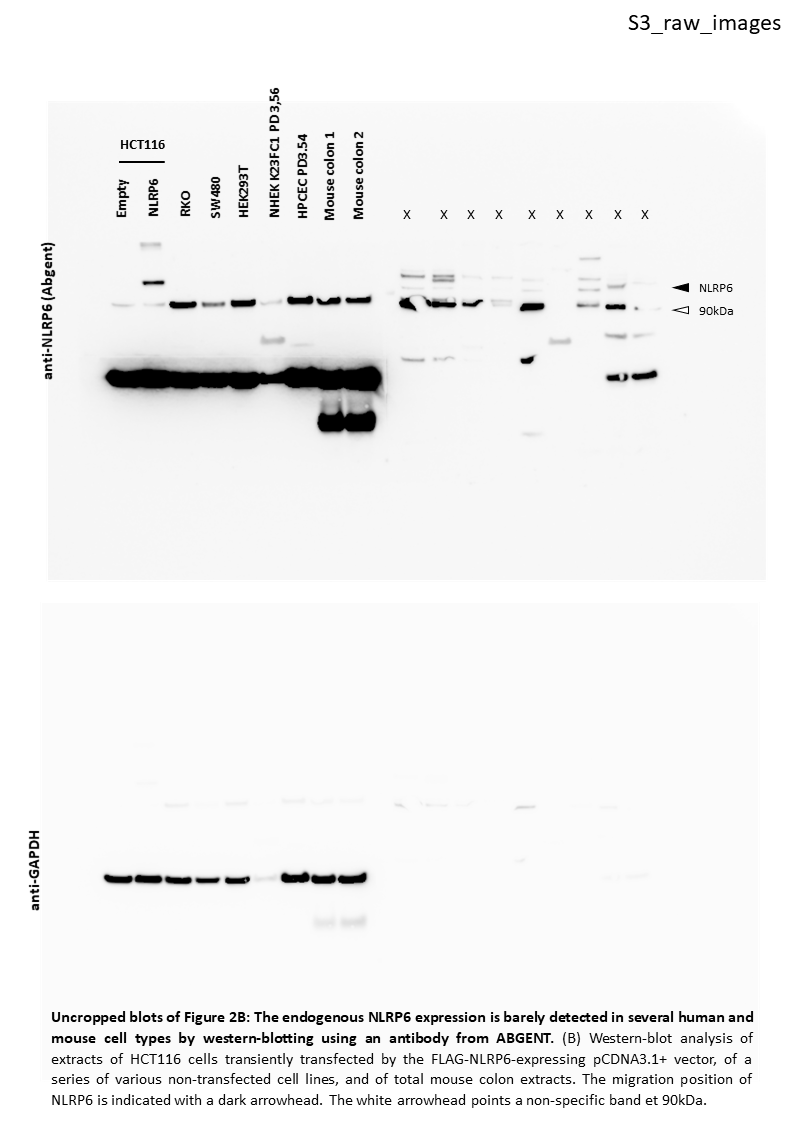

Supplement: S3 Raw images — (TIF) [file pone.0279028.s009.TIF]

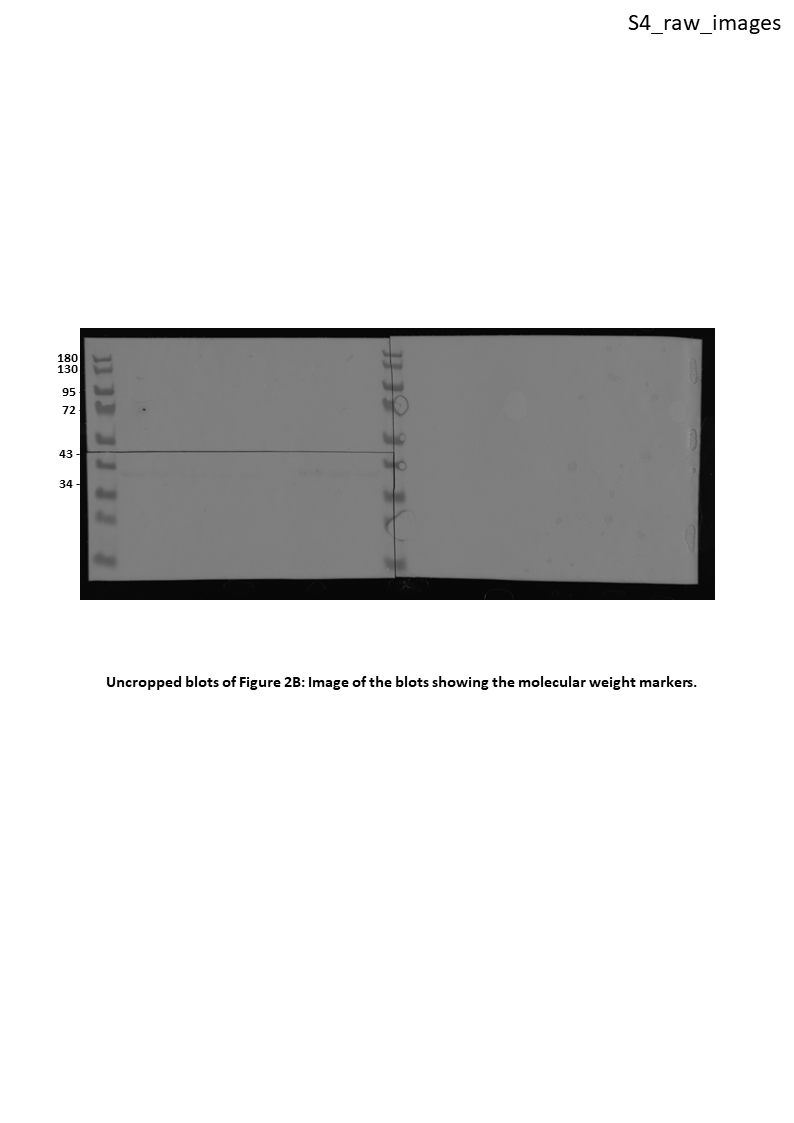

Supplement: S4 Raw images — (TIF) [file pone.0279028.s010.TIF]

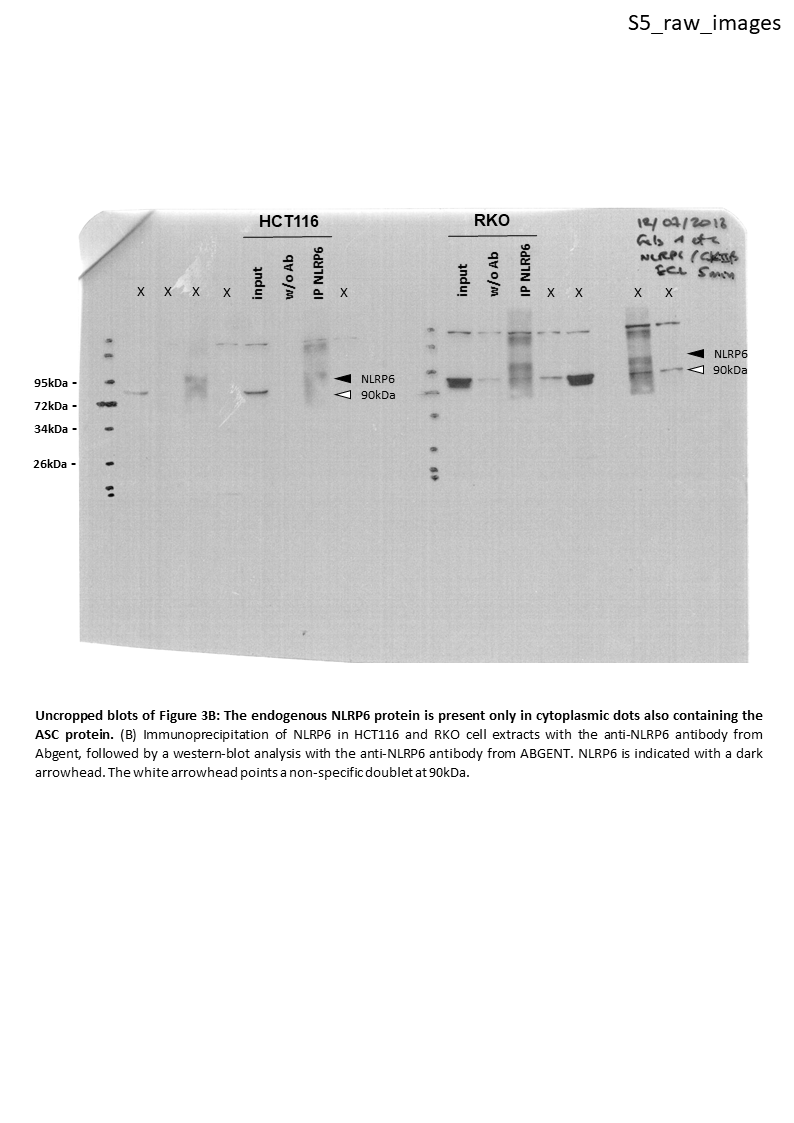

Supplement: S5 Raw images — (TIF) [file pone.0279028.s011.TIF]

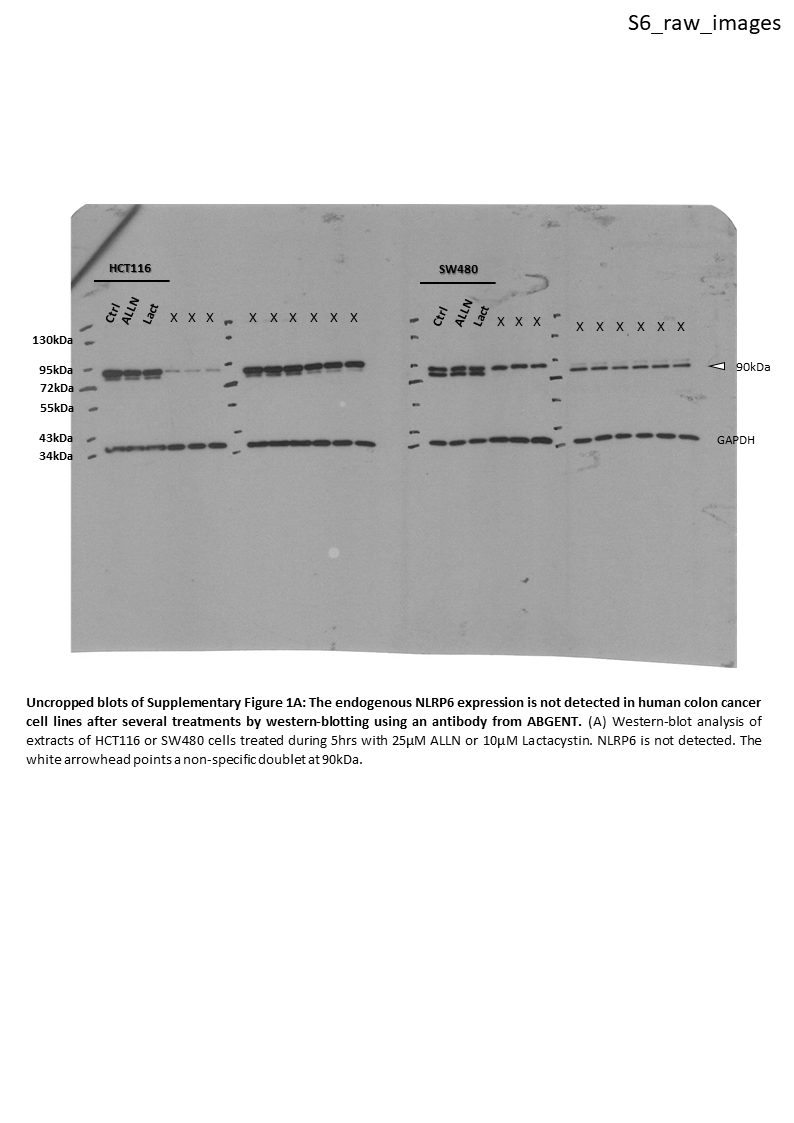

Supplement: S6 Raw images — (TIF) [file pone.0279028.s012.TIF]

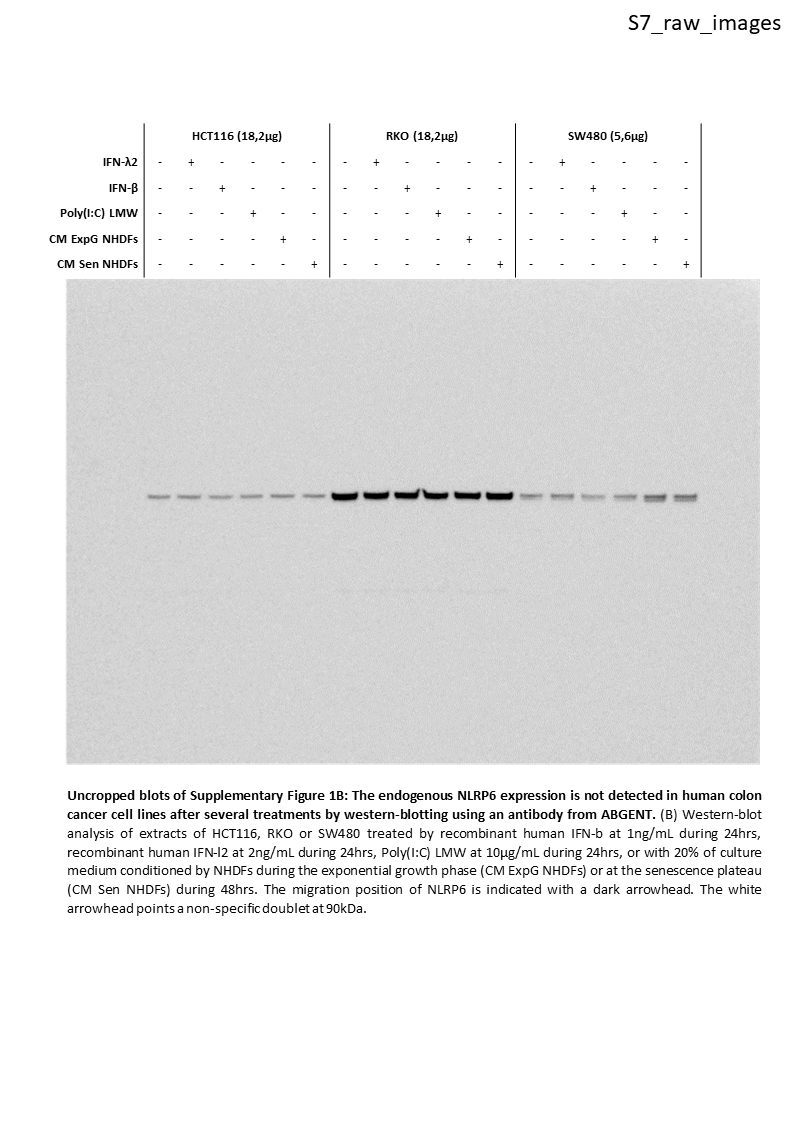

Supplement: S7 Raw images — (TIF) [file pone.0279028.s013.TIF]

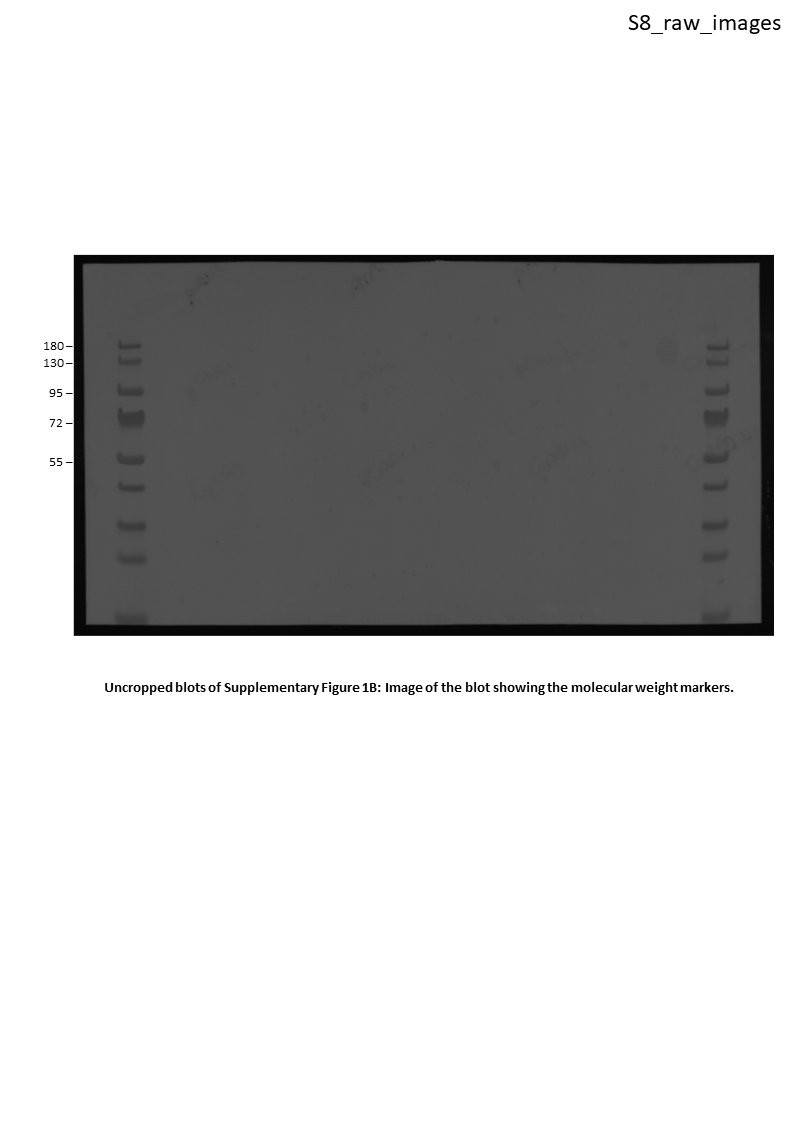

Supplement: S8 Raw images — (TIF) [file pone.0279028.s014.TIF]

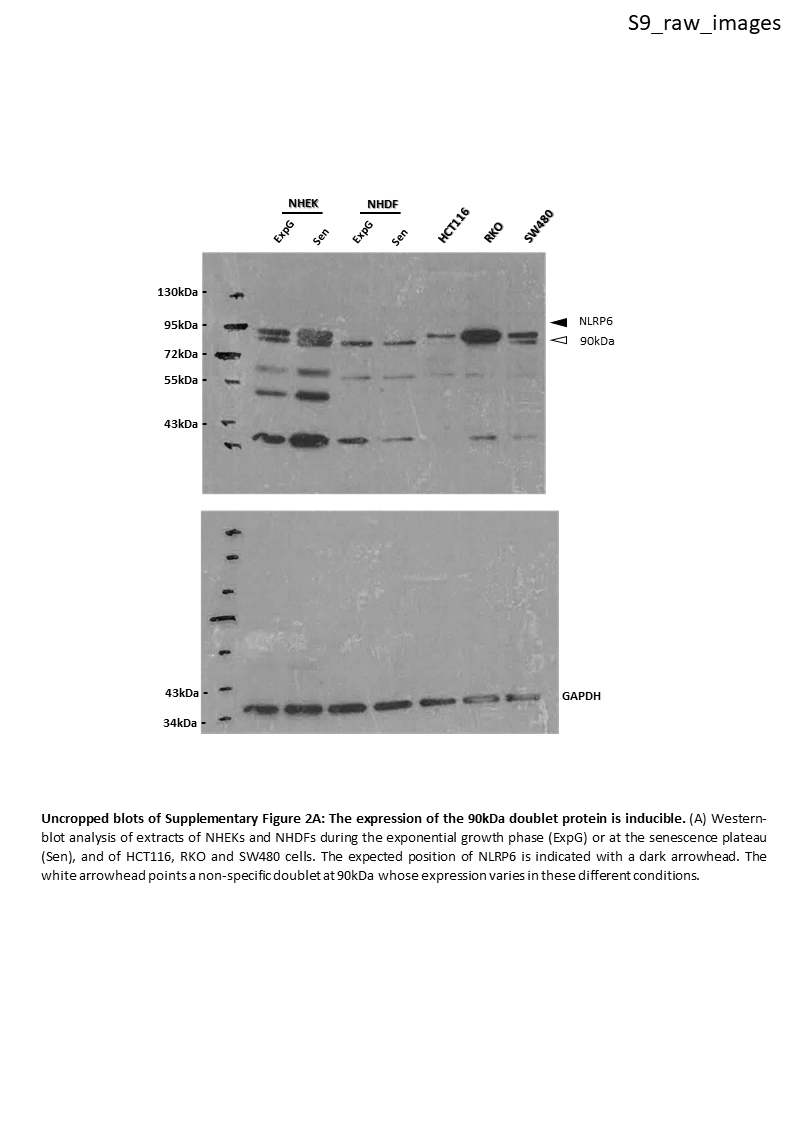

Supplement: S9 Raw images — (TIF) [file pone.0279028.s015.TIF]

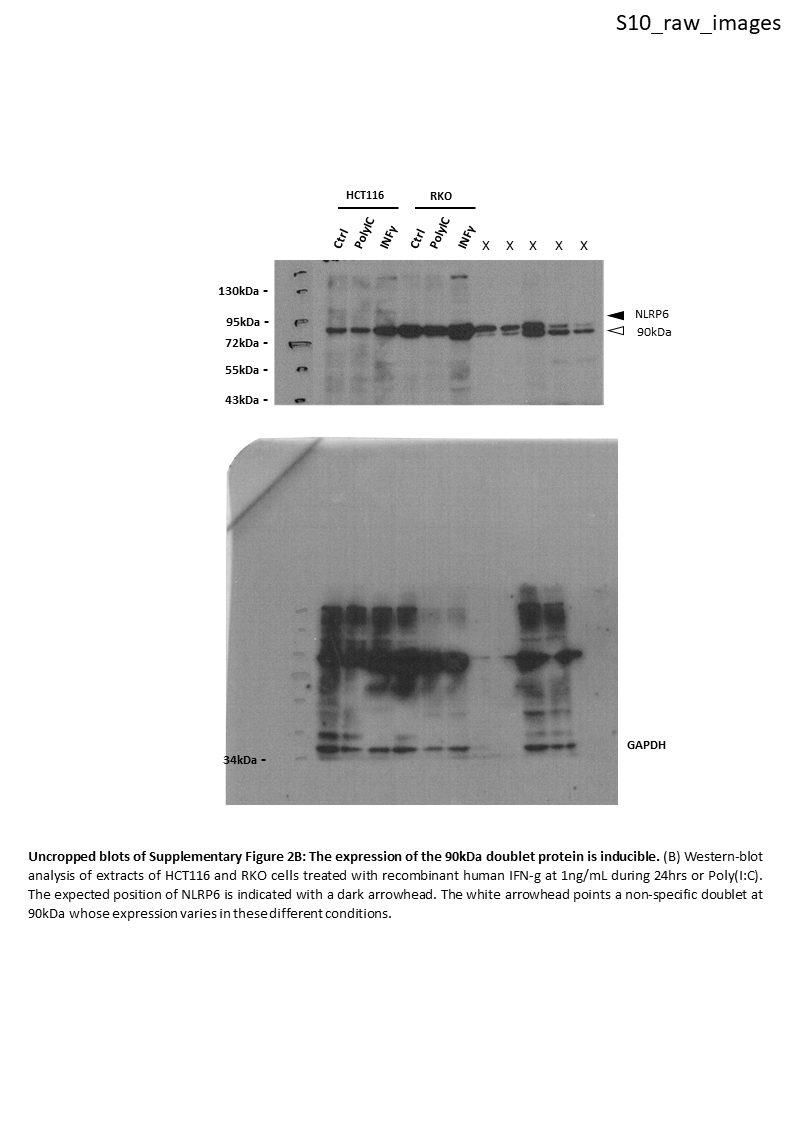

Supplement: S10 Raw images — (TIF) [file pone.0279028.s016.TIF]

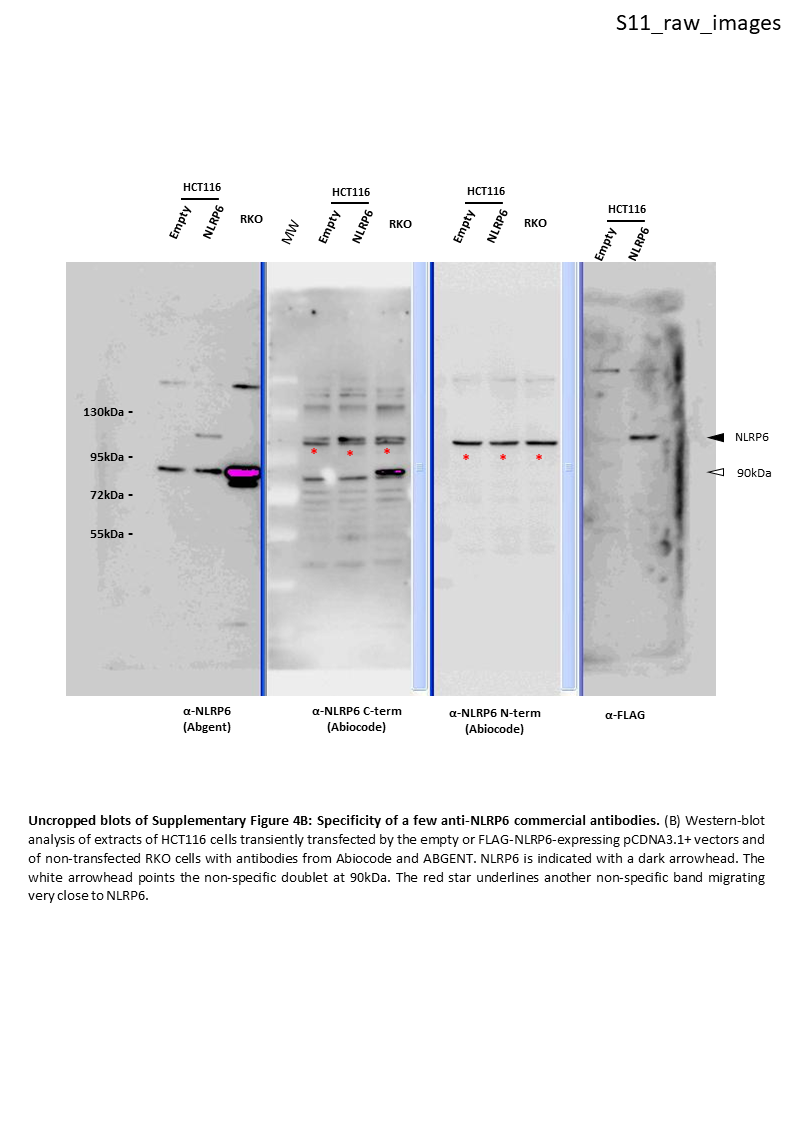

Supplement: S11 Raw images — (TIF) [file pone.0279028.s017.TIF]

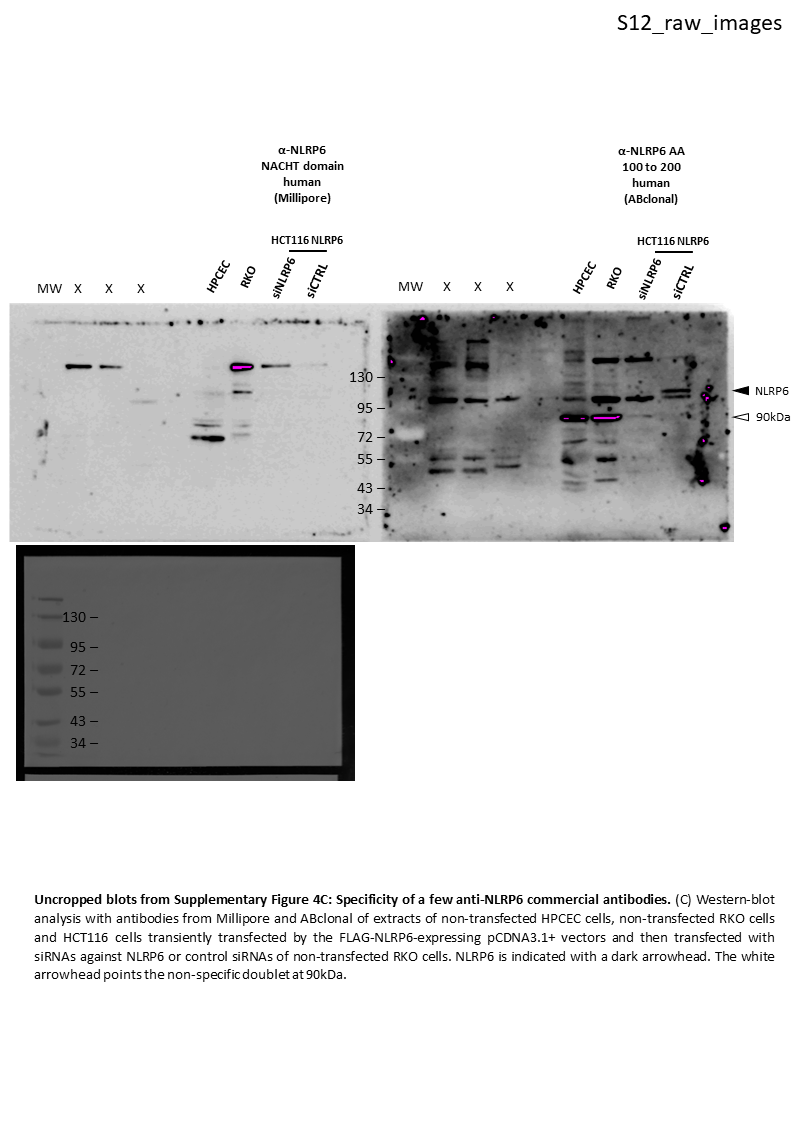

Supplement: S12 Raw images — (TIF) [file pone.0279028.s018.TIF]

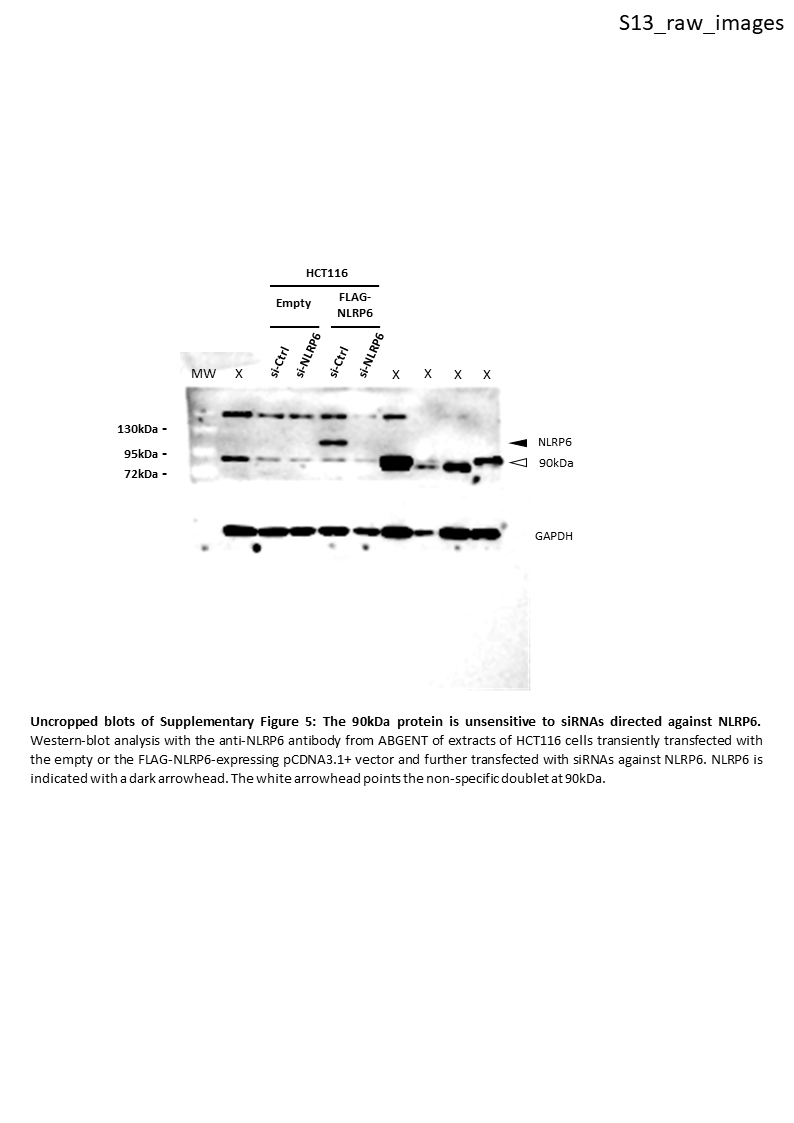

Supplement: S13 Raw images — (TIF) [file pone.0279028.s019.TIF]

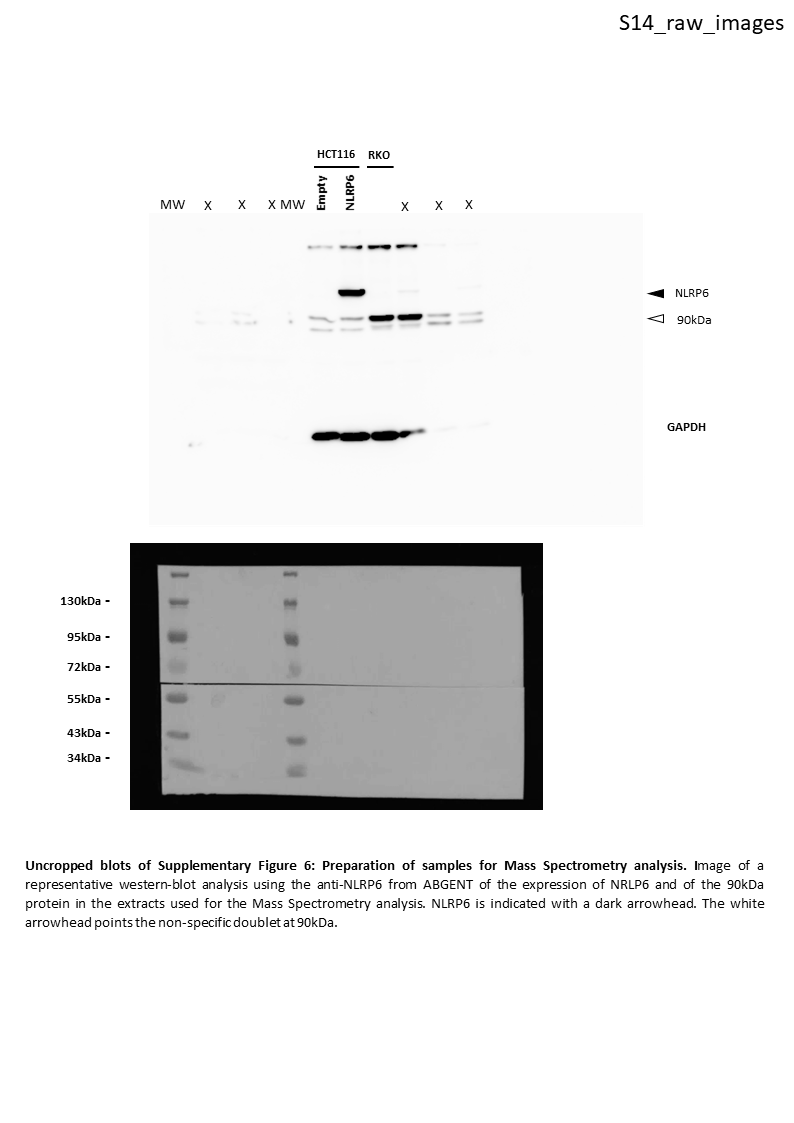

Supplement: S14 Raw images — (TIF) [file pone.0279028.s020.TIF]
